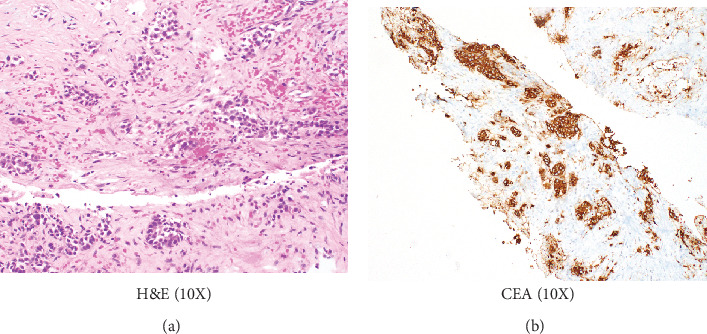# Medullary Thyroid Carcinoma Presenting as Metastatic Disease to the Breast

**DOI:** 10.1155/2020/6138409

**Published:** 2020-05-22

**Authors:** Archana P. Kanteti, Samir Atiya, Ashley Hein, Jesse L. Cox, Ernesto Martinez Duarte

**Affiliations:** Department of Pathology and Microbiology, University of Nebraska Medical Center, USA

## Abstract

Medullary thyroid carcinoma (MTC) is a neuroendocrine tumor that is derived from C cells of the thyroid gland. It is a rare aggressive tumor, known to metastasize to lymph nodes, liver, bones, and lungs. A 41-year-old female, who presented with a breast mass, was initially diagnosed with invasive ductal carcinoma. She was also found to have a thyroid mass which was later diagnosed as MTC. On a rereview of the breast pathology, the morphologic features were strikingly similar to the MTC. Further investigation revealed that this was in fact a very rare case of MTC that had metastasized to the breast. We have identified 20 cases of MTC metastasizing to the breast in the literature that supports its occurrence as a real possibility. Albeit rare, medullary thyroid carcinoma should be considered in the differential diagnosis of a breast mass.

## 1. Introduction

Medullary thyroid carcinoma (MTC) is a neuroendocrine tumor that arises from parafollicular or C cells of the thyroid gland which produce calcitonin. It is a rare neoplasm and known to be quite aggressive and can metastasize to cervical and mediastinal lymph nodes, liver, bones, and lungs (and rarely brain) via lymphovascular spread. It is responsible for 2-3% of all thyroid cancers and usually presents as a painless thyroid nodule [1]. MTCs can be due to either sporadic or hereditary causes. The sporadic form of the tumor is much more commonly encountered and is responsible for 70% of all MTCs. The remaining 30% are due to gain of function germline mutations in the *RET* proto-oncogene inherited in an autosomal dominant fashion [1]. Unfortunately, up to 70% of patients who present with a palpable mass already have cervical lymph node metastases and up to 10% have metastases to distant regions in the body [1]. Some patients with advanced disease can also develop dysphagia and obstructive symptoms due to aggressive local growth [1]. Patients with distant metastasis can also develop flushing and diarrhea due to the elevated levels of circulating calcitonin [1]. Not only is Carcinoembryonic antigen (CEA) a significant tool for follow up but it is extremely useful in cases of MTC which have low-level production of calcitonin [1].

Histologically, MTCs' are made up of nests of neoplastic C cells that can be oval, round, or spindle shaped. These nests are separated by varying amounts of fibrovascular stroma. The tumor cells stain positively for calcitonin and CEA.

MTC metastasizing to the breast is extremely rare, and only 20 cases have been reported in the literature. This case highlights the importance of considering metastatic carcinoma, particularly thyroid as a potential site of primary disease, as part of the differential diagnosis when evaluating a breast mass since the treatment course will change depending on the site of origin. It is also imperative for clinicians to perform a thorough physical exam and work up of the patient.

## 2. Case Presentation

The patient is a 41-year-old woman with no significant past medical history, who presented with a tender right breast lump a few weeks after having a negative routine mammogram. She underwent an ultrasound examination, which showed two separate masses, the largest measuring 1.3 x 1.2 x 0.9 cm. Both lesions were biopsied and subsequently diagnosed as Nottingham Grade 2/3 invasive ductal carcinoma.

**Figure 1:**
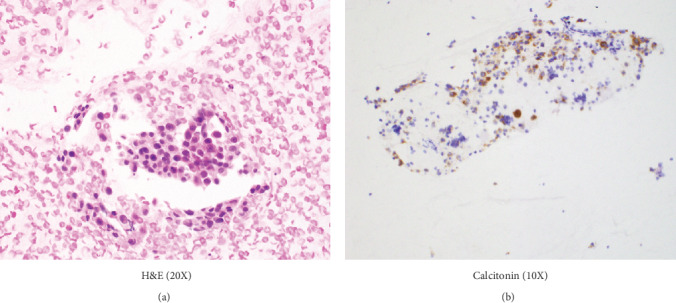


During this time, the patient also noted a neck mass and started complaining of fatigue and altered sleep. An ultrasound of the thyroid gland revealed a 2.4 x 1.6 x 1.4 cm hypoechoic mass in the left lobe. A fine needle aspirate from the thyroid nodule revealed clusters of round and plasmacytoid appearing cells with granular eosinophilic cytoplasm and eccentrically placed nuclei with positive staining for calcitonin (Figure 1(a) and Figure 1(b)) and CEA by immunocytochemistry. The patient was thus diagnosed with primary medullary thyroid carcinoma.

**Figure 2:**
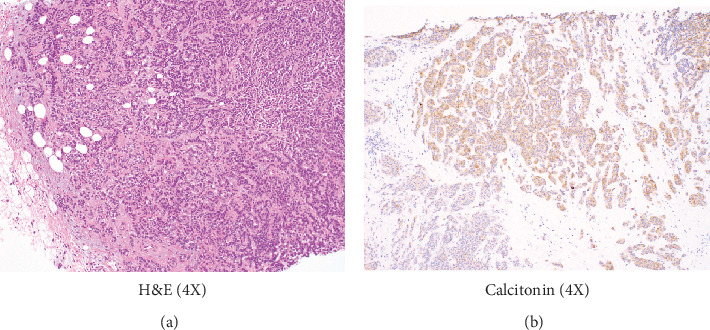


The diagnosis of MTC prompted a rereview of her breast biopsy, with additional immunohistochemical studies. Microscopic examination revealed sheets and nests of hyperchromatic polygonal tumor cells with scant intervening stroma (Figure 2(a). Both breast lesions had a similar morphology to that seen on the thyroid FNA and tested positive for calcitonin (Figure 2(b) and CEA. She underwent total thyroidectomy and central compartment lymph node dissection as well as lumpectomy of the breast masses.

Gross examination of the thyroid revealed a solitary, yellow-tan mass measuring 2.5 cm in the left mid to upper lobe of the thyroid, that appeared to be well-circumscribed and far from the soft tissue edge. Histologically, the lesion was well encapsulated, composed of nests and sheets of polygonal cells, intermediate in size with pale nuclei, dispersed chromatin, and granular cytoplasm. Intratumoral fibrosis was not identified, and lymphovascular invasion was prominent. Immunohistochemistry for calcitonin and CEA were positive in the tumor cells. There was no histologic or gross evidence of extrathyroidal extension. Five out of eight lymph nodes from the central neck dissection were positive for metastasis, and extracapsular extension was present.

CT of the neck, chest, and abdomen was also performed to check for further metastasis. Results showed a 4 mm small noncalcified lung nodule and a 3.6 x 2.5 cm liver lesion. Biopsy of the liver lesion showed nests of hyperchromatic polygonal tumor cells surrounded by dense eosinophilic stroma (Figure 3(a). Immunohistochemistry for CEA was strongly positive in the tumor cells (Figure 3(b). Biopsy of the lung lesion also revealed metastatic medullary thyroid carcinoma, with similar histomorphology to that found in the thyroid and the breast masses.

The patient denied any family history of multiple endocrine neoplasia syndromes, medullary thyroid carcinomas, and breast cancer. Molecular testing for the *RET* proto-oncogene mutation was negative at our institution. A liquid biopsy performed at another institution showed that the tumor cells were positive for a *RET* proto-oncogene mutation. This discrepancy could be due to the initial molecular test not covering the region of the gene that was identified by subsequent testing. This patient's final pathologic stage was IVc (pT1a, pN1a, and pM1) metastatic MTC. She was enrolled in a clinical trial and had been receiving Loxo-292 chemotherapy and a RET inhibitor for the past six months. The patient's symptoms have significantly improved, and there is a 53% decrease in tumor size since the start of the above therapies.

## 3. Discussion

Medullary thyroid carcinoma (MTC) is a rare neuroendocrine tumor that accounts for 1-4% of all thyroid cancers in the United States [2–4]. It originates from parafollicular (C cells) of the thyroid and its pattern of spread is predominantly lymphovascular. Metastasis occurs early to local lymph nodes such as the paratracheal and lateral cervical lymph nodes [3, 5] via lymphatics. Spread outside the neck occurs later in the course of the disease, via venous invasion, commonly to the liver, lungs, and bones with less frequent spread to the skin and brain [3].

Primary breast carcinoma is the second most common malignancy in the female population; second only to skin cancer [6]. Metastasis to the breast from all cancers is an extremely uncommon event, accounting for less than 2% of breast cancers [7]. Most frequently reported metastatic cancers to the breast are malignant melanoma, lymphoma, lung, ovary, prostate, kidney, stomach, ileum, thyroid, and cervical cancer [7, 8].

Metastasis from medullary thyroid carcinoma occurs via lymphatic and blood channels, spreading to cervical and mediastinal lymph nodes and secondarily to the lung, liver, and bones [9]. Metastatic thyroid carcinoma to the breast is extremely rare, with 20 reported cases to our knowledge in the English literature [8]. Nineteen of the twenty cases were reported in females. The interval between thyroidectomy and detection of breast metastasis was between 0-28 years with a mean of 6 years and a mean age at diagnosis of 42.5 years (range 29-72) [8]. Only two of the 20 patients had metastasis to the breast as the initial site of presentation [8].

This further emphasizes the rarity of a metastatic medullary thyroid carcinoma to this anatomic site [10] and might cause diagnostic errors, unnecessary procedures, and unjustified, more aggressive treatment modalities [11] for the patient. The fact that primary breast carcinomas can present with neuroendocrine features, and undergo neuroendocrine differentiation, makes the diagnostic arena even more complicated [12].

Differentiating between metastatic disease to the breast and primary neuroendocrine carcinoma can be extremely difficult, since morphologic and immunophenotypic features would be similar. Neuroendocrine neoplasms tend to have an organoid, nested architecture with high nuclear to cytoplasmic ratio, and salt and pepper chromatin. The presence of necrosis and high mitotic rate does not help in differentiating either primary versus a metastatic process. Both tend to be positive for neuroendocrine markers such as synaptophysin, chromogranin, and CD56 with very few neoplasms expressing other markers specific to their site of origin, like is the case for medullary thyroid carcinoma with positivity for calcitonin [13–17]. Also, primary neuroendocrine carcinomas of the breast would stain negatively for TTF1 and PAX 8. However, medullary thyroid carcinomas can focally express TTF1 and PAX 8 on many occasions [18, 19].

It is important to keep this diagnosis on the differential of breast masses, as treatment and prognosis are significantly different for metastatic thyroid carcinoma as opposed to a primary breast cancer. If misdiagnosed as a primary mammary carcinoma, adequate management can be delayed causing harm to the patient [12].

## 4. Conclusion

We have described here the second case of metastatic MTC presenting without prior history of thyroid disease. Although breast metastasis is a rare occurrence, it should be kept in the differential, as it would radically change the treatment modality for the patient. Any atypical presenting breast cancer cases in a patient with a history of thyroid cancer should prompt work up for metastatic disease. Further research comparing immunohistochemical staining patterns can be useful to further help pathologists differentiate between MTC metastasis versus primary breast cancer.

## Figures and Tables

**Figure 3:**